# Familial multiple endocrine neoplasia type 1 with intrathoracic low-grade fibromyxoid sarcoma

**DOI:** 10.1186/s40792-024-01809-w

**Published:** 2024-01-11

**Authors:** Hiroto Ishida, Soichiro Funaki, Seiji Taniguchi, Eiichi Morii, Yasushi Shintani

**Affiliations:** 1 Department of General Thoracic Surgery, Osaka University Graduate School of Medicine, 2-2, Yamadaoka, Suita-City, Osaka 565-0871 Japan; 2 Department of Thoracic Surgery, Osaka Habikino Medical Center, 3-7-1, Habikino, Habikino-City, Osaka 583-8588 Japan; 3 Department of Pathology, Osaka University Graduate School of Medicine, 2-2, Yamadaoka, Suita-City, Osaka 565-0871 Japan

**Keywords:** Multiple endocrine neoplasia type 1, Low-grade fibromyxoid sarcoma, Thoracic tumor

## Abstract

**Background:**

Multiple endocrine neoplasia type 1 (MEN1) is a hereditary tumor syndrome characterized by endocrine tumors with mainly a parathyroid, pancreatic, or anterior pituitary origin. Low-grade fibromyxoid sarcoma (LGFMS) is a rare low-grade soft tissue tumor. There is one known report of a patient with MEN1 complicated by LGFMS, which is very rare. Our report represents the second documented case, providing valuable insights.

**Case presentation:**

A 31-year-old man with the chief complaint of a cough underwent chest contrast-enhanced computed tomography, which revealed a giant hypoabsorptive tumor with a maximum diameter of 23 cm in the left thoracic cavity. The patient was diagnosed with MEN1, as he also possessed a pancreatic neuroendocrine tumor and parathyroid tumor, and because his father had been found to have MEN1. To control hypercalcemia, surgery for the parathyroid tumor was initially performed, followed by surgical resection of the giant thoracic tumor for diagnosis and treatment. Histopathological examination findings of the tumor resulted in a diagnosis of LGFMS.

**Conclusion:**

We experienced a very rare MEN1 with LGFMS. Although endocrine tumors generally occur more frequently in MEN1, non-endocrine tumors such as the present case should also be noted, reinforcing the importance of systemic imaging scrutiny in addition to early diagnosis and long-term follow-up of MEN1 patients.

**Supplementary Information:**

The online version contains supplementary material available at 10.1186/s40792-024-01809-w.

## Background

Multiple endocrine neoplasia type 1 (MEN1) is an autosomal dominant inherited tumor syndrome characterized by endocrine tumors, mainly with a parathyroid, pancreatic, or anterior pituitary origin. Furthermore, MEN1 is known to be associated with non-endocrine tumors of the central nervous system, skin, smooth muscle, and other mesenchymal tumors, most of which are benign [1]. A low-grade fibromyxoid sarcoma (LGFMS) is a rare type of low-grade soft tissue tumor, with a mixture of fibrous and myxomatous components that accounts for only 0.6% of all soft tissue sarcomas [2], most often occurring within skeletal muscles and rarely in the thoracic cavity [3]. There is only one known report of MEN1 with LGFMS [4], which is very rare. Our report represents the second documented case, providing valuable insights.

## Case presentation

**Fig. 1:**
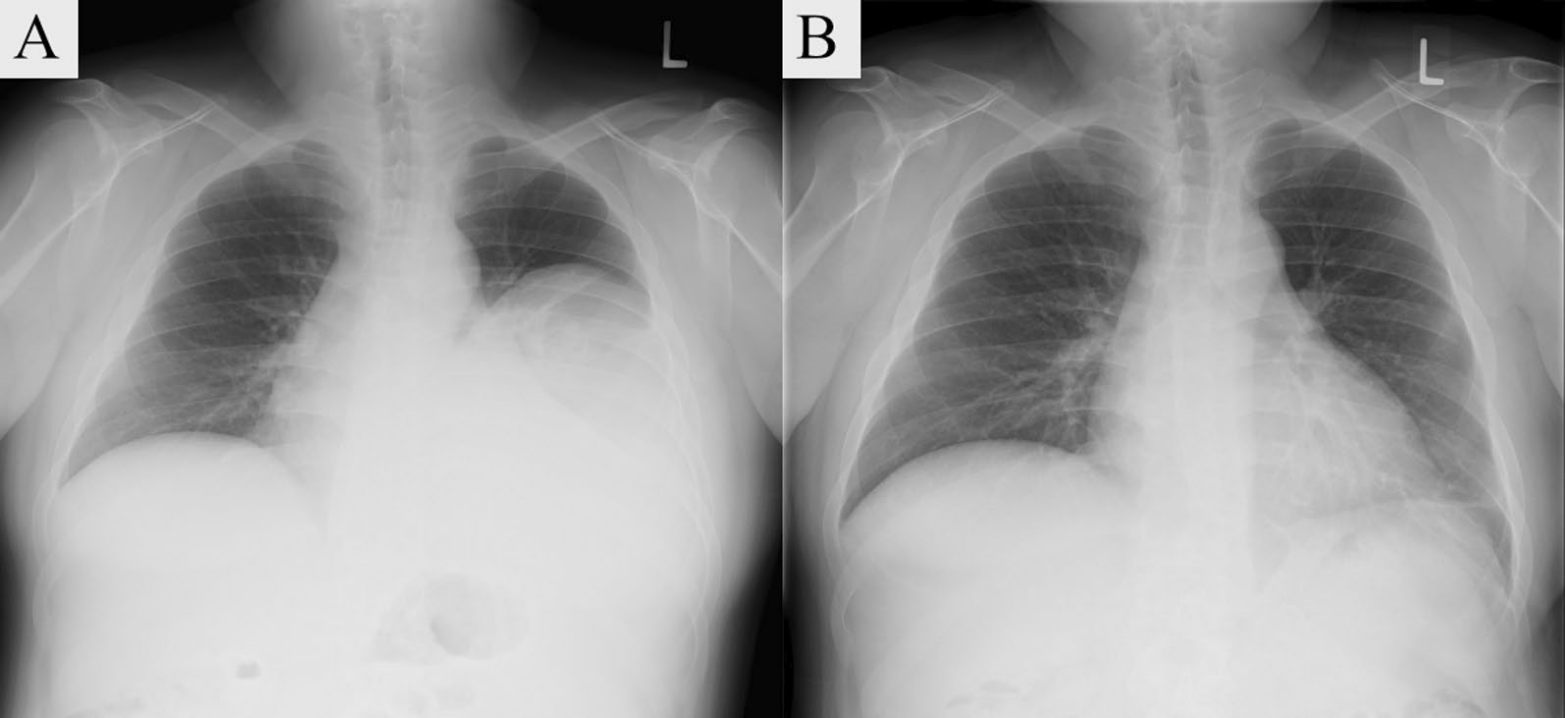
Chest X-ray images. **A** Preoperative image showing decreased permeability in the left middle and lower lung fields. **B** Postoperative image showing that the decreased transparency in the left lung field has disappeared and the expansion is good

**Fig. 2:**
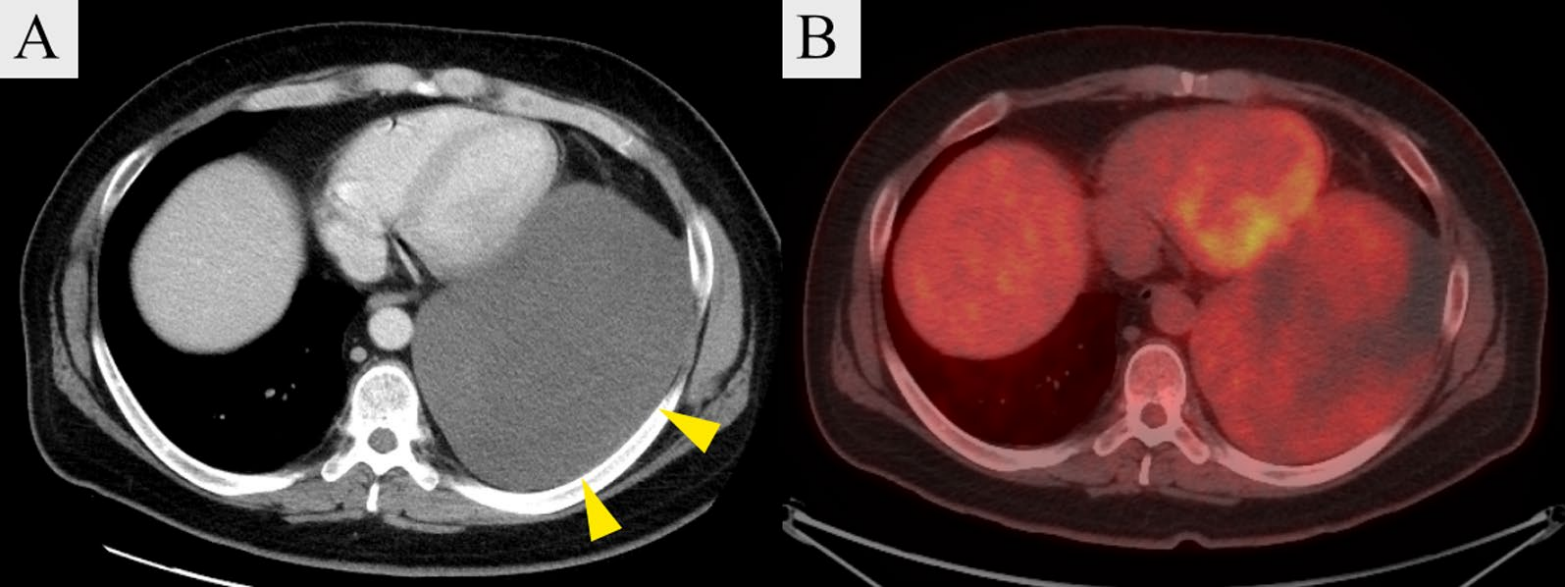
**A** Chest contrast-enhanced computed tomography image showing giant hypoabsorptive tumor with a maximum diameter of 23 cm in the left thoracic cavity, and compressing the left lower lobe and heart. **B** F-18 fluorodeoxyglucose (FDG) positron emission tomography image showing heterogeneous FDG uptake (SUVmax = 3.3). Yellow triangles in **A** indicate the tumor

The patient was a 31-year-old man with a chief complaint of cough, while his medical history and comorbidities included diabetes mellitus, hyperparathyroidism, urinary tract stones, chronic kidney disease, pancreatic glucagonoma, and duodenal gastrinoma. Physical examination findings revealed obesity with a BMI of 34 and blood testing indicated mildly elevated inflammatory response, hypercalcemia, mild renal dysfunction, and a high glycohemoglobin level. Chest X-ray showed decreased permeability in the left middle and lower lung fields (Fig. 1A). Chest contrast-enhanced computed tomography (CT) was performed, which revealed a giant hypoabsorptive tumor with a maximum diameter of 23 cm in the left thoracic cavity compressing the left lower lobe and heart (Fig. 2A), while F-18 fluorodeoxyglucose (FDG) positron emission tomography (PET) showed heterogeneous FDG uptake consistent with the same area (SUVmax = 3.3) (Fig. 2B). Based on these imaging findings, the giant tumor in the left thoracic cavity was suspected to be a chronic expanding hematoma, solitary fibrous tumor, or neurogenic tumor. As for other complications, the patient possessed a pancreatic neuroendocrine tumor and parathyroid tumor along with hyperparathyroidism. In addition, a family history showed that his father suffered from MEN1. Although genetic testing could not be performed due to a lack of patient consent, *MEN1* gene mutations are not essential for a diagnosis of MEN1 [5], and based on the clinical findings and familial information, a diagnosis of MEN1 in the present case was determined.

To control hypercalcemia, resection of the parathyroid tumor was initially performed. Three weeks later, surgical resection of the giant thoracic tumor was performed for diagnosis and treatment, during which stand-by extracorporeal membrane oxygenation (ECMO) was prepared for intraoperative circulatory instability. First, the 5th intercostal space was opened and a huge tumor covered with a white capsule was observed in the thoracic cavity, leading to suspicion of a solitary fibrous or neurogenic tumor. Because of the large size, it was difficult to obtain a clear view, thus the 6th rib was removed and an additional chest opening was made in the 7th intercostal space. The tumor had relatively sparse inflammatory adhesions to the chest wall, while adhesions near the vertebral body on the caudal side of the tumor were strong, indicating a pleural tumor arising from this site. There was no grossly apparent bony invasion, and the pleura was dissected to the vicinity of the vertebral body, and the tumor was removed. Post-extraction left lower lobe re-expansion was good and there was no evidence of reperfusion injury (see Additional file 1).

**Fig. 3:**
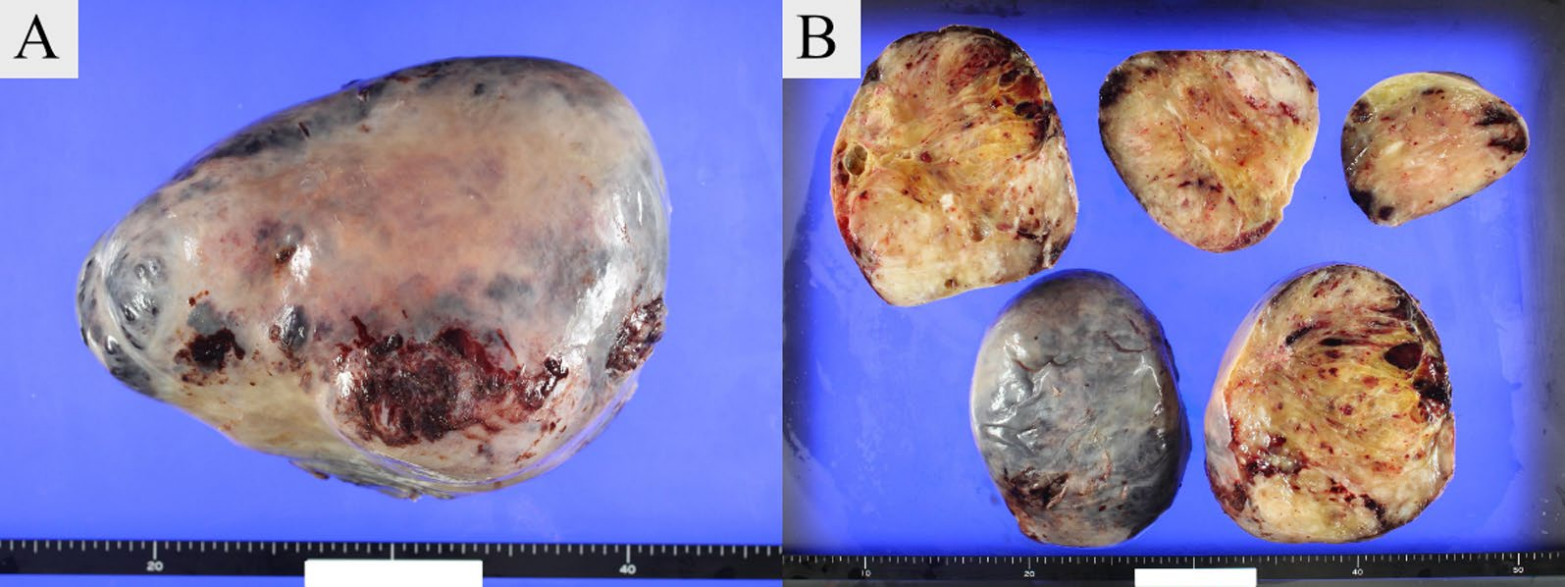
Macroscopic appearance of tumor. **A** A yellowish-white mass surrounded by a capsule, **B** with internal septal structures and mucus

**Fig. 4:**
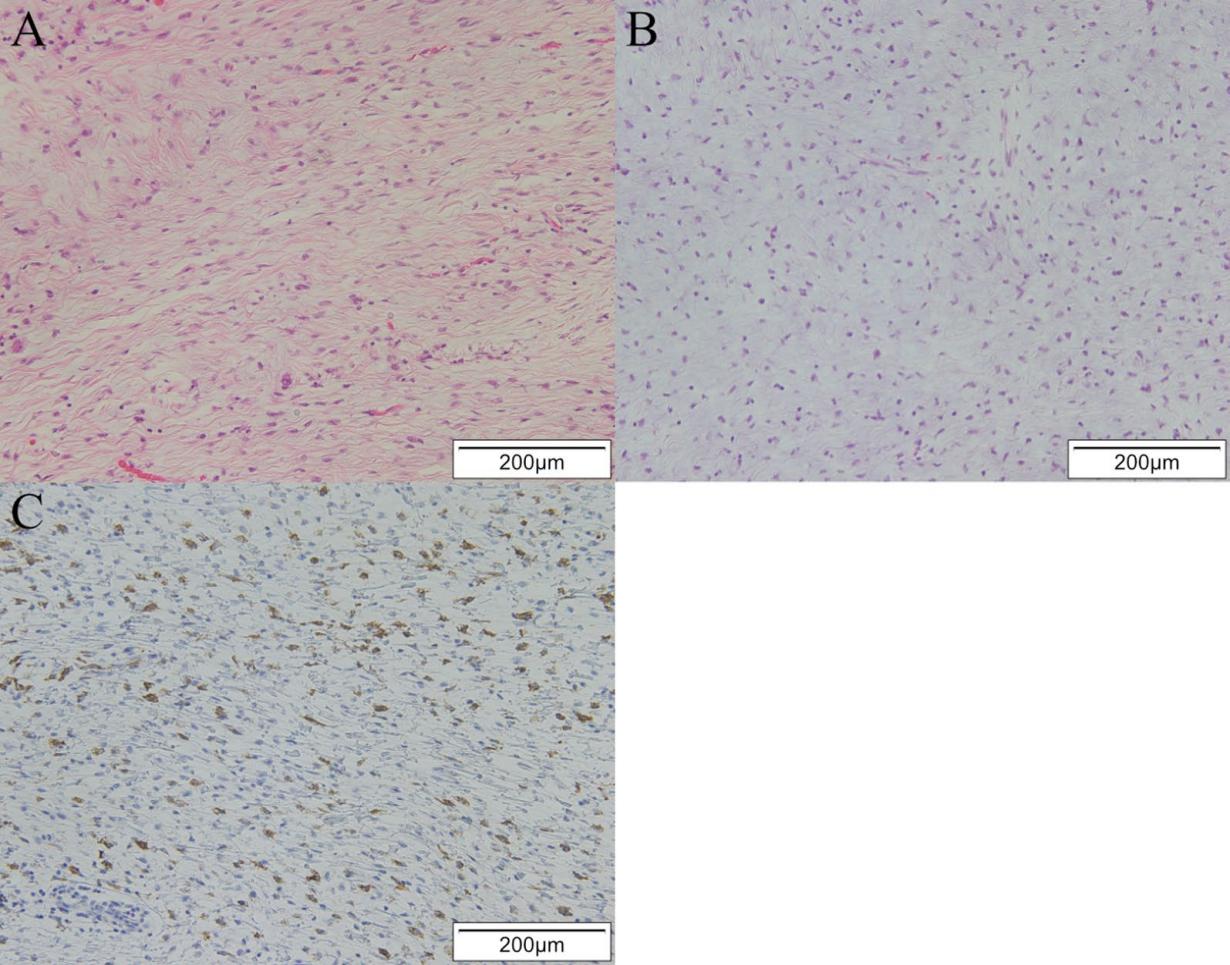
Histological imaging. Hematoxylin–eosin images (× 200) showing spindle-shaped tumor cell proliferation, with a mixture of **A** fibrous and **B** mucus components. **C** Mucin 4 was positive with immunostaining (×200)

The excised tumor was 25 cm in its greatest diameter and weighed 3 kg. Macroscopic findings showed a yellowish-white mass surrounded by a capsule, along with internal septal structures and mucus (Fig. 3A, B). Hematoxylin–eosin imaging indicated a spindle-shaped tumor cell proliferation, with a mixture of fibrous and mucus components (Fig. 4A, B). Immunostaining was negative for epithelial, vascular, lymphatic, and neural markers, and positive for alpha-smooth muscle actin (αSMA), a smooth muscle marker. The Ki-67 proliferation index was 1% and positive findings for mucin 4, a marker noted in LGFMS cases and related mesenchymal tumors [6], were noted (Fig. 4C). These findings led to a diagnosis of LGFMS, and the resection margins were negative. Postoperative lung expansion was good (Fig. 1B) and the patient was discharged without complications. At three years after surgery, there was no evidence of disease, and we will continue to follow the patient every 6 months, including any local recurrence.

## Discussion

MEN1 is an inherited tumor syndrome characterized by endocrine tumors, mainly with a parathyroid, pancreatic, or anterior pituitary origin, with a wide variety of tumors known to occur in affected individuals throughout life. However, it is difficult to predict which types are likely to develop because of no correlation between genotype and phenotype [1]. The effects of MEN1 on affected patients depends on complications associated with malignancy, and early detection and treatment lead to improved prognosis. Therefore, an asymptomatic diagnosis based on genetic testing findings and long-term follow-up examinations are very important [7–10]. However, the awareness of MEN1 in Asia including Japan is lower than in Europe and the United States, and genetic testing for such asymptomatic diagnosis is not widely performed [11]. The present patient was estranged from his father who had a history of MEN1, thus he did not undergo regular examinations and the disease was discovered in association with a large lesion occupying the thoracic cavity. As he had previously been diagnosed with and treated for relatively young onset diabetes and multiple urinary tract stones associated with hypercalcemia, it is highly likely that MEN1 could have been diagnosed and treated earlier had detailed systemic examinations been performed at that time. As for the future, conducting long-term surveillance in accordance with the guidelines [5] is important.

LGFMS is a low-grade soft tissue tumor with a mixture of fibrous and myxomatous components that occurs mainly within the skeletal muscles of the limbs and trunk, and rarely in the thoracic cavity. Therefore, it is difficult to identify LGFMS preoperatively in thoracic tumor cases [3]. Imaging findings such as low to moderate absorption in CT scanning [12] are typical, and helpful for diagnosis. While FDG-PET typically shows relatively faint FDG uptake [13], cases without FDG uptake have been reported [2]. Findings obtained with preoperative imaging in this case were consistent with LGFMS, though a differential diagnosis was not considered and, unexpectedly, LGFMS was demonstrated by the histopathological results. Although MEN1 is known to be associated with a variety of tumors, only a single case of LGFMS with MEN1 has been reported [4]. LGFMS is not known as a common complicating tumor, making preoperative differentiation in the present case difficult. In addition, non-endocrine tumors associated with MEN1 are often benign [1] and intraoperative findings, which showed the mass to be covered by a capsule, presented no evidence of surrounding invasion or extensive adhesions, leading us to strongly suspect that the tumor was benign. Treatment for LGFMS is based on complete resection, which has been shown to be associated with a good long-term prognosis [14–16]. On the other hand, there are cases of local recurrence and postoperative irradiation may be considered when the resection margin cannot be secured [17]. The resection margins in this case were negative, and postoperative irradiation was not performed.

Various non-endocrine tumors associated with MEN1 have been reported, including facial angiofibroma [18], lipoma [18], meningioma [19], leiomyoma [20], and breast cancer [21], but only one case of MEN1 with LGFMS has been reported. As for the only reported case of LGFMS with MEN1 [4], that patient had no family history of MEN1, thus it was most likely a sporadic occurrence, indicating that the present is the first reported case of LGFMS with familial MEN1. The authors of that previous case report raised a question regarding the lack of correlation between genotype and phenotype in LGFMS, because their patient had sporadic MEN1 and a novel genetic mutation was detected. While the present patient was diagnosed with familial MEN1 based on complications associated with an endocrine tumor and family history, he did not wish to undergo genetic testing, thus the possible presence of *MEN1* gene mutations or mutation patterns was not examined. This is a limitation in our case report. However, although this case was a familial MEN1, the father of our patient had no history of LGFMS, supporting no correlation between genotype and phenotype even in LGFMS cases.

## Conclusion

We experienced a very rare MEN1 with LGFMS. Although endocrine tumors generally occur more frequently in MEN1, non-endocrine tumors such as the present case should also be noted, reinforcing the importance of systemic imaging scrutiny in addition to early diagnosis and long-term follow-up of MEN1 patients.

### Supplementary Information

**Additional file 1: **Surgical video.

## Data Availability

Not applicable.

## Abbreviations

LGFMS: Low-grade fibromyxoid sarcoma
MEN 1: Multiple endocrine neoplasia type 1
CT: Computed tomography
FDG: Fluorodeoxyglucose
PET: Positron emission tomography
ECMO: Extracorporeal membrane oxygenation
αSMA: Alpha-smooth muscle actin
